# Avoidance of recognition sites of restriction-modification systems is a widespread but not universal anti-restriction strategy of prokaryotic viruses

**DOI:** 10.1186/s12864-018-5324-3

**Published:** 2018-12-07

**Authors:** I. S. Rusinov, A. S. Ershova, A. S. Karyagina, S. A. Spirin, A. V. Alexeevski

**Affiliations:** 1 Belozersky Institute of Physical and Chemical Biology, Lomonosov Moscow State University, 119992 Moscow, Russia; 2 Gamaleya National Research Center of Epidemiology and Microbiology of the Ministry of Health of the Russian Federation, 123098 Moscow, Russia; 3 All-Russia Research Institute of Agricultural Biotechnology, 127550 Moscow, Russia; 4 Faculty of Bioengineering and Bioinformatics, Lomonosov Moscow State University, 119991 Moscow, Russia; 5 National Research University Higher School of Economics, 101000 Moscow, Russia; 6 Institute of System Studies, 117281 Moscow, Russia

**Keywords:** Restriction-modification systems, Anti-restriction, Site avoidance, Compositional bias, Bacteriophages, Archaeal viruses

## Abstract

**Background:**

Restriction-modification (R-M) systems protect bacteria and archaea from attacks by bacteriophages and archaeal viruses. An R-M system specifically recognizes short sites in foreign DNA and cleaves it, while such sites in the host DNA are protected by methylation. Prokaryotic viruses have developed a number of strategies to overcome this host defense. The simplest anti-restriction strategy is the elimination of recognition sites in the viral genome: no sites, no DNA cleavage. Even a decrease of the number of recognition sites can help a virus to overcome this type of host defense. Recognition site avoidance has been a known anti-restriction strategy of prokaryotic viruses for decades. However, recognition site avoidance has not been systematically studied with the currently available sequence data. We analyzed the complete genomes of almost 4000 prokaryotic viruses with known host species and more than 17,000 restriction endonucleases with known specificities in terms of recognition site avoidance.

**Results:**

We observed considerable limitations of recognition site avoidance as an anti-restriction strategy. Namely, the avoidance of recognition sites is specific for dsDNA and ssDNA prokaryotic viruses. Avoidance is much more pronounced in the genomes of non-temperate bacteriophages than in the genomes of temperate ones. Avoidance is not observed for the sites of Type I and Type IIG systems and is very rarely observed for the sites of Type III systems. The vast majority of avoidance cases concern recognition sites of orthodox Type II restriction-modification systems. Even under these constraints, complete or almost complete elimination of sites is observed for approximately one-tenth of viral genomes and a significant under-representation for approximately one-fourth of them.

**Conclusions:**

Avoidance of recognition sites of restriction-modification systems is a widespread but not universal anti-restriction strategy of prokaryotic viruses.

**Electronic supplementary material:**

The online version of this article (10.1186/s12864-018-5324-3) contains supplementary material, which is available to authorized users.

## Background

Bacteria and archaea have a range of defense systems that protect them from viral infections [1]. Restriction-modification (R-M) systems are a kind of innate immunity of prokaryotes that is widely spread among both bacteria and archaea. R-M systems protect prokaryotic cells from an invasion of foreign DNA, particularly viral DNA [2]. A typical R-M system includes a restriction endonuclease (REase) and a methyltransferase (MTase). The REase recognizes short DNA sequences, called recognition sites (RS), and then cleaves the DNA. The MTase modifies the same sites in the host DNA and thus protects them from the REase activity (for a review see [3] for additional details on R-M system functioning and types). The defense functions of R-M systems makes them meaningful players in co-evolution of prokaryotes and their viruses.

A reduction in the number of RS in a viral genome increases the probability of overcoming the defense provided by an R-M system. This was directly demonstrated for λ *cI857* bacteriophage and the EcoRI R-M system [4]. Thus it is reasonable to expect that natural selection would often result in a decreased number of RS in genomes of prokaryotic viruses. An elimination of RS in the genome of coliphage fd under R-M system pressure was demonstrated in laboratory experiments in 1967 [5].

An under-representation of RS was confirmed for dozens of genomes of prokaryotic viruses (bacteriophages and archaeal viruses) by comparing the number of sites with its statistical expectation [6–8]. RS avoidance is regarded as one of strategies of prokaryotic viruses to counteract host R-M systems [9]. Notably, RS avoidance was not observed in all studied cases.

RS avoidance is not a specific feature of phage genomes. It has also been observed in genomes of prokaryotic organisms [8, 10, 11]. The counterselection against RS in prokaryotic genomes is caused by the imperfect self/non-self discrimination, which could occasionally lead to autoimmunity due to self-DNA cleavage [12]. Avoidance in prokaryotic genomes has been comprehensively investigated [13].

R-M enzymes are divided into four main types (Type I, Type II, Type III, and Type IV) and a range of subtypes according to their subunit composition, biochemical properties, cofactor requirements, and other features [14]. In accordance with a number of studies [15, 16], we designate Type II R-M systems excluding subtypes IIG and IIM “orthodox Type II R-M systems”. Orthodox Type II R-M systems consist of independently acting REase and MTase proteins. We have previously shown that only orthodox Type II systems provoke RS avoidance in prokaryotic genomes [13].

RS avoidance in the genomes of prokaryotic viruses has yet not been systematically described. The last investigation on the subject provided an analysis of avoidance of palindromes in several dozen phage genomes available at that time (2001) [8]. The limited data on phage genomes and R-M system specificities did not allow the authors to verify some interesting suggestions or to analyze several aspects of RS avoidance. In particular, it is still not clear how common this strategy is among the viruses, whether it is equally effective against R-M systems of different types, or if it can be used simultaneously with other anti-restriction mechanisms. The current work is aimed at a comprehensive investigation of RS avoidance in the genomes of prokaryotic viruses using a method that we previously used to study avoidance in prokaryotic genomes [13, 17]. We analyzed the representation of RS of host-encoded R-M systems in the complete genomes of prokaryotic viruses with different genome types and lifestyles. We demonstrated that RS avoidance is a specific feature of DNA viruses and that it is more characteristic for non-temperate bacteriophages. Additionally, only RS of orthodox Type II R-M systems are commonly avoided in genomes of prokaryotic viruses. The avoidance of RS of R-M systems of the other types seems to be either unnecessary or ineffective.

## Methods

### Genome sequences

A list of complete genomes of bacteriophages and archaeal viruses was obtained through Entrez requests to the NCBI Nucleotide database [18] as described by Grazziotin and co-authors [19]. The list was supplemented with two lists from the ENA Genome Pages for bacteriophages and archaeal viruses [20] and with several dsRNA bacteriophage genomes from GenBank that were missed using the previous methods. All currently known dsRNA bacteriophages belong to the family Cystoviridae. These bacteriophages are the only prokaryotic viruses with segmented genomes that have been discovered so far. The missing genomes were obtained by cross-referencing from the Cystoviridae page of NCBI Taxonomy.

The resulting list includes 4996 genomes (see Additional file 1). To reduce taxonomical bias, we selected only the longest genome for each unique Taxonomy ID (in NCBI databases one Taxonomy ID can be assigned to slightly different genomes if they are regarded as one strain). However, some bacteriophages with identical or nearly identical genomes have different Taxonomy IDs. Therefore, we also grouped bacteriophages with higher than 99% genomic sequence identity and obtained a single member from each group. As a result, 3407 representative genomes (marked in Additional file 1 with “yes” in “Reference” column) were selected for the subsequent analysis.

Among the studied prokaryotic viruses are eight ssDNA and 81 dsDNA archaeal viruses. All of the other 3318 prokaryotic viruses have bacterial hosts, i.e., are bacteriophages. In particular, all RNA prokaryotic viruses are bacteriophages. Therefore, we use terms “bacteriophage” or “phage” instead of “prokaryotic virus” in most cases in this text.

We used genomes of eukaryotic viruses as a control set because such viruses do not encouter R-M systems during their life cycle (with the exception of particular viruses of green algae [21]), and their genomes are close to the phage genomes in size. Eukaryotic virus genomes are also abundant in nucleotide databases. These genomes were obtained from the ENA [20]. We chose only the longest sequence among entries with the same Taxonomy ID. Thus, only the longest segment was taken from segmented genomes of the eukaryotic viruses, unlike the segmented prokaryotic viruses (dsRNA Cystoviridae phages). The control set of eukaryotic viruses includes 4021 sequence entries (see Additional file 2).

### Viral lifestyles

The duration of persistence of a viral genome within the host cell is important for our purposes. According to Hobbs and co-authors [22], we differentiate between temperate and non-temperate bacteriophages. Unfortunately, all data on phage lifestyles are fragmentary and ambiguous: lytic, lysogenic, and virulent bacteriophages do not correspond exactly to “temperate” or “non-temperate” terms [22]. Thus we roughly treat lytic and virulent bacteriophages as non-temperate bacteriophages, lysogenic bacteriophages and prophages as temperate bacteriophages.

We obtained lifestyle information from the annotations of the NCBI Nucleotide records of the viral genomes, including references in these annotations. We additionally used the lists of lytic bacteriophages (treated as non-temperate in this study) and temperate bacteriophages from PhAnToMe SEED [23]. As a result we were able to annotate 435 temperate bacteriophages and 394 non-temperate bacteriophages (see Additional file 3, column “Lifestyle”). The obtained annotations could be erroneous in individual cases but appears to be sufficient for generalized statistical assessments.

### Viral hosts

Viral hosts were determined using the annotations of the viral genomes and the related literature. However, the prokaryotic strains specified as viral hosts often have no complete genome sequences available and thereby no data on encoded R-M systems. Therefore, we extended the host range up to species to be able to match the R-M systems with the phages. We presumed that a phage has an increased probability of being exposed to those R-M systems that are encoded in the genomes of any strains of its host species.

We assigned hosts to 3274 bacteriophages (see Additional file 3, column “Hosts”). There are 417 different host species, and R-M systems are annotated in the genomes of 231 of them. In some cases several host species were assigned to a single phage.

### Restriction-modification systems

We downloaded information about all 17,896 REases from R-M systems with annotated (verified or predicted) specificity encoded in the genomes of 2930 bacterial and archaeal species from the REBASE [24, 25] (see Additional file 4). This list of species includes all 231 abovementioned hosts of bacteriophages and archaeal viruses.

### Datasets of (site, genome) pairs

We regard pairs consisting of a phage genome and a recognition site of a REase encoded in a genome of at least one of the phage hosts. For each such pair, the site is expected to be under the selective pressure in the phage genome because of the activity of the REase. Our Experimental dataset includes 66,704 such pairs (see Table 1 and Additional file 5). Only RS of length greater than 2 were regarded. This dataset was to detect evolutionary pressures of host R-M systems on RS numbers.

**Table 1:** Composition of Experimental dataset with respect to Types of R-M systems recognizing the site. The same site could be recognized by R-M systems of different Types, as shown in the last three rows

Type of REase	Number of (site, genome) pairs	Number of different sites	Number of different genomes
I	23,223	202	1571
II	34,912	186	2767
IIG	2584	65	1081
IIM	975	6	628
III	3795	43	1050
IV	597	2	597
II and IIG	425	5	399
II and IIM	167	3	167
II and III	26	1	26

It is important to note that the numbers from Table 1 cannot be directly compared with the data on abundance of REases of different types (see, for example, Oliveira et al. [26]). Our set includes only REases with annotated RS longer than 2 bp.

We constructed two negative control datasets. Control dataset 1 consists of pairs of each of all 899 different RS of REases and each representative genome from Additional file 1 (bacteriophages). Control dataset 2 consists of pairs of the same sites and all genomes from Additional file 2 (eukaryotic viruses). Control dataset 1 contains the entire Experimental dataset, which, however, constitutes only a minor part of it, namely 2.2% (66,704 of 3,062,893). Table 2 contains a comparison of site-genome composition of the datasets used in this work.

**Table 2:** Composition of the datasets used in this work

Dataset	Number of different sites	Number of different genomes	Number of (site, genome) pairs
Experimental dataset	494^a^	2861^b^	66,704
Control dataset 1	899	3407	3,062,893
Control dataset 2	899	4021	3,614,879

^a^R-M systems encoded in the genomes of the known phage hosts recognize 494 among all 899 known RS. ^b^Only 2861 phages among 3407 have known host species with available data on the encoded R-M systems

We assume that control sets, consisting of natural sequences that are mostly not exposed to R-M systems, are preferred over simulated sequences because the natural sequences of phages and viruses may have some common features, such as density of coding regions, affecting oligonucleotide composition.

### Compositional bias calculation

We used the method of Burge and co-authors [27] to detect under-represented RS in the genome sequences. This method implies calculation of so-called compositional bias (CB), which is the ratio of the observed to the expected frequency of a site of interest. The expected frequency is estimated based on the observed frequencies of all subsites of a given site. The same procedure has been previously used to investigate RS avoidance in prokaryotic genomes [13], and has a higher accuracy than Markov chain-based methods [17, 28].

The CB value should be equal to 1 if there is no selective pressure on the site in the genome. A CB value less than 1 could indicate a selection against the site. We used the threshold 0.8 to consider a deviation significant. We call a site *under-represented* if its CB value is less than 0.8 in the corresponding genome and at the same time the expected number of sites is greater than 15. We also used a CB threshold of 0.1 for extreme under-representation, and such sites are considered *eliminated* in this work.

## Results

### Influence of viral genome type on RS avoidance

R-M systems generally only target double-stranded DNA molecules. However, we also analyzed compositional bias values for RNA and single-stranded DNA bacteriophages. In Table 3 we present only the data for palindromic Type II RS in prokaryotic (Experimental dataset and Control dataset 1) and eukaryotic (Control dataset 2) viruses with different types of genomes. The analogous tables for the other Types of R-M systems and for asymmetric Type II RS are presented in Additional file 6. These tables contain some values that could indicate significant site under-representation. However, they either share the trends of Table 3 (see below) or appear to be independent of the presence of R-M systems.

**Table 3:** Percentages of RS with compositional bias value (CB) less or greater than 1

Genome type	Experimental dataset		Control dataset 1		Control dataset 2	
	CB < 1	CB > 1	CB < 1	CB > 1	CB < 1	CB > 1
dsDNA^a,b^	85.3% (22678)	14.6% (3883)	69.3% (335436)	30.6% (148037)	53.5% (70490)	46.2% (52069)
ssDNA^a,b^	68.6% (1294)	31.1% (586)	60.8% (11868)	38.8% (7577)	51.8% (51409)	47.7% (40962)
ssRNA	55.9% (208)	43.5% (162)	53.1% (1072)	46.6% (941)	51.3% (160372)	48.3% (151175)
dsRNA	50.9% (27)	49.1% (26)	46.5% (532)	53.1% (608)	52.3% (6402)	47.3% (5790)

^a^The experimental set significantly differs from Control set 1 (*p*-value < 0.01, Fisher’s exact test)

^b^The experimental set significantly differs from Control set 2 (*p*-value < 0.01, Fisher’s exact test)

**Fig. 1:**
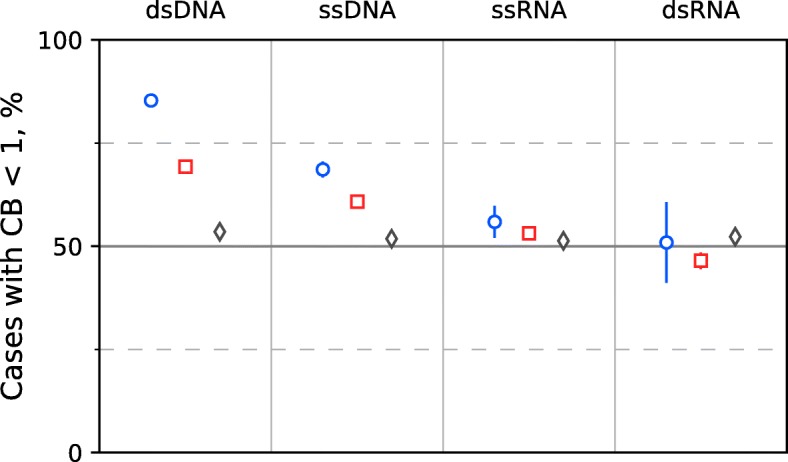
Percentages of sites with reduced numbers of occurrences in the viral genomes of different types. Blue circles are for the Experimental dataset, red squares are for Control dataset 1 (prokaryotic viral control), and gray diamonds are for Control dataset 2 (eukaryotic viral control)

The fractions of RS with a reduced observed frequency (CB < 1) differ significantly among all three datasets (one experimental and two controls) in the case of dsDNA and ssDNA viruses, but this is not in the case for dsRNA and ssRNA viruses (Table 3, Fig. 1). This clearly indicates the existence of a selective pressure on RS in the genomes of DNA bacteriophages but not in the genomes of RNA phages. The significant difference between the fractions with CB < 1 and CB > 1 in Control dataset 1 cannot be completely explained by the inclusion of the Experimental dataset. This could mean the presence of a large number of bacteriophages that meet the corresponding R-M systems, but we have no data regarding these interactions. The slight deviation of the eukaryotic viral control fractions from 50% could be caused by some functional role of 4–6 bp palindromic sites unrelated to the activity of R-M systems.

The observed avoidance in case of ssDNA bacteriophages could reflect R-M system influence during the double-stranded stage of their lifecycle. It should be noted that the fractions of RS with CB < 1 reliably differ between dsDNA and ssDNA phages. Only dsDNA phages were selected for the subsequent analysis because of insufficient data for ssDNA phages in the majority of the studied cases.

### REase type effect on RS avoidance

**Fig. 2:**
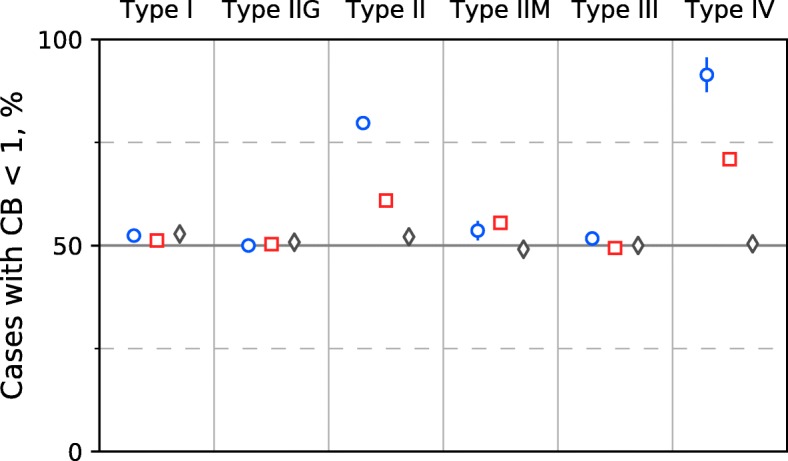
Percentages of RS with reduced numbers of occurrences calculated for the different types of R-M systems. Designations are the same as in Fig. 1

We separately analyzed Type I, Type IIG, orthodox Type II, Type IIM, Type III, and Type IV REases. For this purpose, we split our datasets by REase type. The substantially increased fractions of RS with reduced counts (CB < 1) were observed only for two REase types: orthodox Type II and Type IV (see Fig. 2). Note that, in case of Type IV, this is true only for CB < 1 but not for under-representation (CB < 0.8, see the next paragraph).

**Fig. 3:**
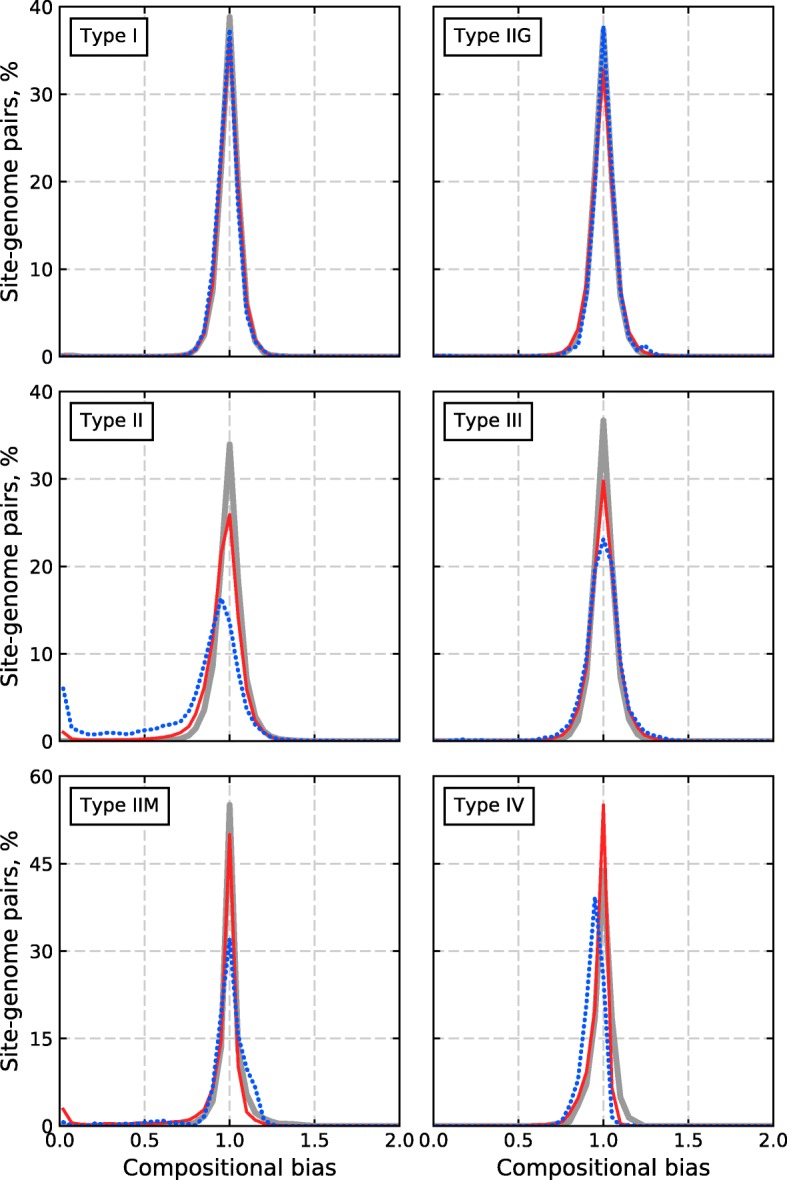
Histograms of compositional bias values for different types of R-M systems. Dotted blue lines correspond to the subsets of the Experimental dataset, solid red lines are for Control dataset 1, and gray solid lines are for Control dataset 2

We calculated histograms of CB values for the subsets of the experimental and control datasets for different R-M system types (Fig. 3). The histograms demonstrate that only the Type II subset is significantly enriched for under-represented sites. Namely, there are 27.2% under-represented sites of orthodox Type II R-M systems and fewer than 5% of such sites for other types of R-M systems. The fraction of eliminated (CB < 0.1) RS is 7.1% for the Type II R-M systems and less than 0.5% for the R-M systems of the other types.

Our experimental dataset include a significant fraction of predicted RS which were not experimentally verified. Erroneous annotations of RS may result in decreased fractions of under-represented RS. However, the fractions of eliminated and under-represented sites for proved RS are similar to the fractions for predicted RS. There are 34.5 and 3.3% under-represented sites among proved RS of Type II and the other types, correspondingly. The analogous fractions of eliminated sites among proved RS are 7.1 and 0.3%, respectively.

In the cases of Type I and Type IIG RS, there is no significant difference between the distributions of CB values for the experimental and control datasets (Figs. 2 and 3). Thus, there is likely no selective pressure against Type I and IIG sites in the phage genomes, or at least such pressure is not strong and stable enough to induce RS avoidance.

For Type III RS, the difference between the experimental and control distributions is almost undistinguished on Figs. 2 and 3. However, the fractions of Type III sites with CB < 1 and CB < 0.8 significantly differ between the experimental and each of the control datasets (*p*-value< 0.008 in all cases, Fisher’s exact test). This is due to a minor fraction (approximately 5%) of Type III sites that are under-represented in the phage genomes. Nevertheless, RS avoidance does not seem to be a considerable anti-restriction strategy in the case of Type III R-M systems. Note that the fractions of over-represented Type III sites also significantly differ in the experimental and the control datasets.

The histogram for the Type IIM subset of the Experimental dataset (Fig. 3) demonstrates that the fraction of sites with CB > 1.1 are slightly, but significantly, larger than the corresponding fractions of both control datasets (*p*-value< 1e-10 in each case, Fisher’s exact test). The list of Type IIM RS includes nine palindromic and five asymmetric sites. The percentage of (genome, asymmetric site) pairs in the Type IIM fraction of the Experimental dataset is only 6.0%, and thus the asymmetric sites cannot significantly affect the observed histogram. All Type IIM palindromic sites are also recognition sites of some orthodox Type II R-M systems. The fractions of genomes with CB < 1 vary significantly for different sites: 100% for GATC, 88.5% for GCNNGC, 66.3% for GCWGC, and only 22.5% for GCNGC (five other known Type IIM RS are not present in our Experimental dataset). The site GCNGC exhibits pairs that form a bulge at the CB~ 1.1 area of the histogram (Fig. 3). Such an overabundance of GCNGC sites in the phage genomes seems to be unrelated to R-M systems because the fractions with CB > 1 for GCNGC are also large in both controls (60.7% in Control dataset 1 and 93.6% in Control dataset 2).

The majority (91.4%) of Type IV sites have CB < 1, but for the most of them CB is close to 1 (see Fig. 3). There are only two different Type IV RS that meet our criteria, SCNGS and YCGR. Both sites are rather small, GC-rich and have strong intersections with the common Type II sites CCNGG and CCGG, respectively. The control dataset of eukaryotic viruses, unlike the control dataset of bacteriophages, contains almost identical fractions of Type IV sites that are more frequent and less frequent than expected. This suggests that the observed difference might be related to R-M system activity.

### Avoidance of RS in genomes of phages with different lifestyles

**Fig. 4:**
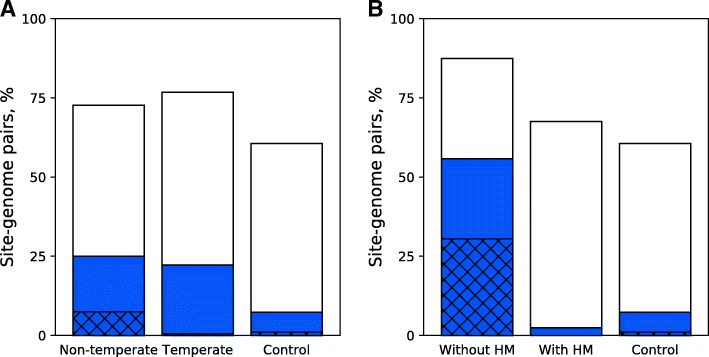
Fractions of Type II sites with different CB values. Bar height corresponds to the fraction of sites with the reduced number (CB < 1), the colored portion is for sites with CB < 0.8, and the hatched portion indicates the fraction with CB < 0.1. “Control” stands for the subset of Control dataset I with Type II sites and dsDNA genomes. **a** Comparison of temperate and non-temperate dsDNA viruses. **b** Comparison of the coliphages with or without the hydroxymethylase (HM) gene

We compared histograms of CB values for orthodox Type II RS in genomes of temperate and non-temperate DNA bacteriophages (Fig. 4a). Fractions of sites with reduced (CB < 1) and significantly reduced (CB < 0.8) numbers of occurrences are similar for temperate and non-temperate phages. However, temperate phages have a notably lower fraction of RS that are eliminated from their genomes (CB < 0.1). Such differences may be because of the prophage period, during which an extreme bias of oligonucleotide composition does not give any selective advantage.

### Phages with anti-restriction mechanisms

We estimated how other anti-restriction mechanisms could influence RS avoidance. We used coliphages of the Myoviridae family as a convenient example. Some phages of the family encode an anti-restriction enzyme (DNA hydroxymethylase), which modifies the genomic DNA and thus prevents DNA cleavage by REases. Other related phages do not encode this enzyme [29]. Figure 4b shows that five phages with such an anti-restriction mechanism avoid (CB < 0.8) only 2.4% of actual *E. coli* RS, while 55.8% of such sites are avoided in genomes of four coliphages of the same family that do not encode the DNA hydroxymethylase.

## Discussion

RS avoidance is not a universal anti-restriction strategy for prokaryotic viruses. Only 3.9% of our Experimental dataset are excluded sites (CB < 0.1) and 17.7% are under-represented sites (CB < 0.8). To determine the avoidance constraints we examined RS avoidance in bacteriophage genomes with respect to various characteristics of the bacteriophages and R-M systems encoded in the host genomes.

Both single-stranded and double-stranded RNA bacteriophages do not have any noticeable signs of RS under-representation (see Fig. 1). This is exactly as expected because of the specificity of R-M systems to DNA molecules. It also agrees well with previously known data on preferential avoidance of palindromes in genomes of DNA bacteriophages [8].

REases typically do not cleave single-stranded DNA. However, we observed a sign of avoidance of the RS of orthodox Type II R-M systems in the genomes of ssDNA bacteriophages (see Table 3). This likely indicates that ssDNA bacteriophages are susceptible to R-M systems at the double-stranded stage of their lifecycle, which is obligatory for such bacteriophages [8]. Therefore we discuss only RS avoidance in genomes of dsDNA bacteriophages.

We did not observe any elimination or even under-representation of RS of Type I and Type IIG R-M systems, and we found only a few cases of under-represented sites of Type III systems. The observed difference of site representation between those R-M systems and the orthodox Type II R-M systems can be explained by their structural features. Namely, orthodox Type II R-M systems have separate DNA recognition domains for the REase and the MTase. This substantially complicates any change of specificity of such systems providing enough time for phages to reduce their numbers of sites. R-M systems of other types have one recognition domain for both MTase and REase, which facilitates specificity changes. We suggest that the different rates of specificity changes could be the main cause of the observed difference in avoidance of RS for different types of R-M systems.

Another possible reason may be a consequence of much higher genetic diversity of Type II R-M systems in comparison to Type I and Type III systems [3]. Bacteriophages might have a greater chance to develop a universal anti-restriction mechanism against Type I or Type III systems. Such a mechanism could protect from the majority of such systems regardless of their specificity. For example, phage T7 encodes Ocr protein, which can inhibit Type I R-M systems by mimicking the DNA shape and charge [30]; phage T3 has a hydrolase of SAM [31], a ligand essential for the activity of Type I and Type III REases, but not of orthodox Type II REases.

The REase components of Type I and Type III systems can not act regardless of the cognate MTase. This can affect the site under-representation in prokaryotes but not in viruses. In prokaryotic cells, lack of a Type II MTase caused by, for example, expression noise, can result in an accidental cleavage of host DNA. This hypothesis has some experimental support [12]. That is not the case for Types I and III, and this may explain differences in site avoidance in prokaryotic genomes (see further). However, this explanation can not be applied to the case of bacteriophage genomes because, unlike prokaryotic genomes, they are not protected from restriction even in case of normal MTase concentration.

There is another feature of Type I and Type III R-M systems that could influence their site avoidance. Unlike Type II R-M systems, Type I and Type III R-M systems translocate along the DNA after site recognition and in most cases hydrolyze the DNA when meeting an analogous complex translocating in the opposite direction [3]. That is why such R-M systems often require two sites that are located in head-to-head orientation to cleave the DNA. Thus bacteriophages have an additional opportunity to overcome the host defense, excluding RS from only one of the DNA strands. Bearing in mind the possibility of such strand-asymmetrical avoidance of RS, we separately considered non-palindromic sites on both DNA strands. Despite the previously known specific examples [32], we did not observe any systematic site avoidance for Type I and Type III R-M systems, including strand-asymmetrical avoidance (see Figs. 2 and 3).

Type IIG systems are in many respects intermediate between Type I and orthodox Type II systems, and our data show that they are close to Type I systems in features that are essential for RS avoidance. Namely, Type IIG systems, as well as Type I and Type III systems, contain the only target recognition domain that allows them to change their specificity much more often than orthodox Type II R-M systems do.

Type IIM and Type IV systems do not contain MTases and include only modification-dependent REases. While protecting the host from phages with modified genomes, the modification-dependent REases are nearly harmless for viruses without such modifications. We observed only a weak RS avoidance for the modification-dependent REases. Considering the fact that all recognition sites of such REases are either also known RS of orthodox Type II R-M systems or have a very strong intersection with them, we hypothesize that the observed weak avoidance is mainly caused by the activity of Type II R-M systems.

With respect to R-M system Types, RS avoidance in the genomes of dsDNA bacteriophages has features similar to the previously shown avoidance in prokaryotic genomes. Only RS of orthodox Type II R-M systems are commonly avoided in both prokaryotic genomes [13] and the genomes of their viruses. Two exceptions for this rule are the sites GATC (Type IIM) and CAGAG (Type III). Both of these sites are avoided in bacterial genomes [13]. At the same time, the Type IIM site GATC has CB < 1 in genomes of all 46 bacteriophages whose hosts encode corresponding REases, and 45 of them have CB < 0.8. The site CAGAG is under-represented in 25 bacteriophages and is among the sites that constitute the small fraction of under-represented Type III RS in the Experimental dataset.

There is a notable difference of RS avoidance between prokaryotes and their viruses. Namely, bacteriophages have a significantly increased fraction of nearly excluded sites compared to prokaryotes. This difference might be explained by the smaller size of the viral genomes compared to the prokaryotic genomes. However, this is not a result of an increased fraction of unreliable CB values for the viral genomes because we only considered (phage, site) pairs with expected numbers of sites exceeding 15. Thus the difference between the phage and prokaryotic genomes is trustworthy. Nonetheless, to reach an extreme CB value a bacterium requires more mutations and thereby it takes longer. This may explain the decreased fraction of sites with CB < 0.1 in the prokaryotic genomes. However, genome size can not be the only reason because the fraction of almost excluded (CB < 0.1) sites is only increased for non-temperate dsDNA bacteriophages, while temperate phages avoid RS much more similarly to the host prokaryotic genomes. Note that temperate phages spend a significant part of their lifecycle as prophages. Thus the discussed difference seems to be mainly caused by the influence of MTases, which weaken the selective pressure against RS in the genomes of temperate phages and prokaryotes.

Temperate phages cannot be considered typical parasites, as the lysogenic infection could be beneficial for a prokaryote [33]. Some bacteria even have certain mechanisms that allow them to preferentially restrict lytic infections. Particular CRISPR/Cas systems have been discovered that can specifically tolerate a lysogenization of the host while preventing a lytic cycle progression [34]. Existence of any similar distinguishing mechanism in the case of R-M systems would clearly explain the observed difference of extreme RS avoidance in genomes of temperate and non-temperate phages. However, a recent attempt to detect such a mechanism failed [35]. Moreover, it is difficult to imagine how such a distinguishing characteristic might function in the case of typical R-M systems that lack complex regulatory components. Thus we assume that the certain tolerance of prokaryotes to temperate phages can not explain the difference in RS avoidance between temperate and non-temperate bacteriophages.

To some extent, our findings contradict the previous comparison of palindrome avoidance between phages and their hosts [8]. It was shown that phages avoid 4 bp and 6 bp palindromes to a lesser extent than the host bacteria. However, this conclusion was based on average abundance of all palindromes of the same length regardless of presence of the corresponding R-M systems in the host genome. Thus the increased fraction of eliminated sites (CB < 0.1) in the genomes of non-temperate phages seems to be masked by other palindromic sites of the same length. Another possible explanation is that the previous comparison was made on a limited set of phages and bacteria that could have some specific trends of RS avoidance.

The lack of RS in a phage genome guarantees protection from cleavage by corresponding REases. This is why we separately considered “site elimination” (CB < 0.1), which clearly represents a strong anti-restriction strategy of phages. We observed site elimination in only 7.5% of cases (Fig. 4a). However, this percentage is likely an underestimate because, following our methodology, we excluded 1191 (phage, site) pairs for which the observed number of sites is zero but the expected number is too low (< 15).

Moderate site under-representation (0.1 < CB < 0.8) indicates a selective pressure against the site. We observed such site under-representation in 17% of our Experimental dataset. In Control dataset 2, CB < 0.8 was observed in only 1.8% of the cases. Moderate site under-representation facilitates the success of a phage attack on the bacterial population. The lower the number of sites, the higher the probability that all sites in a phage genome will be methylated before its cleavage. In that case the genomes of the ancestors of this phage will be methylated and protected from cleavage; see Enikeeva et al. [36] for a mathematical model of the host-phage interaction and Pleška et al. [4] for an experimental proof of a correlation between the number of RS and the fraction of bacteriophages that escape restriction.

Many other anti-restriction mechanisms have been discovered [3, 9, 37]. If a phage evolves some of them then the selective pressure against RS will expectedly weaken. We show that if cytosines in the phage DNA are hydroxymethylated then no site avoidance can be detected. Presumably, the non-temperate dsDNA bacteriophages without a detectable under-representation of orthodox Type II RS are either those that evolved another anti-restriction strategy (such as cytosine hydroxymethylation) or those in contact with the R-M system for too short a time to develop under-representation of its site.

## Conclusions

RS avoidance is a widespread anti-restriction strategy of bacteriophages. However, it is far from a universal strategy. Avoidance is mainly specific to sites of orthodox Type II R-M systems and to dsDNA and ssDNA non-temperate phages that have had contact with the corresponding R-M system for a long enough time and have not evolved another anti-restriction strategy.

## Additional files

Additional file 1: List of complete genomes of prokaryotic viruses. (TSV 344 kb)

Additional file 2: List of selected sequences of eukaryotic viruses. (TSV 376 kb)

Additional file 3: List of prokaryotic viruses. (TSV 304 kb)

Additional file 4: List of REases with known recognition sites. (TSV 467 kb)

Additional file 5: Experimental dataset. (TSV 3578 kb)

Additional file 6: Percentages of sites with CB values less and greater than 1 for different types of R-M systems and phage genomes. (PDF 306 kb)

## Abbreviations

CB: compositional bias
dsDNA: double-stranded DNA
dsRNA: double-stranded RNA
HM: DNA hydroxymethylase
MTase: DNA methyltransferase
REase: restriction endonuclease
R-M system: restriction-modification system
RS: recognition sites
ssDNA: single-stranded DNA
ssRNA: single-stranded RNA
