# Supplementary material for: Avoidance of recognition sites of restriction-modification systems is a widespread but not universal anti-restriction strategy of prokaryotic viruses
Source: BMC Genomics. 2018 Dec 7;19:885. doi: 10.1186/s12864-018-5324-3 (PMC6286503; doi:10.1186/s12864-018-5324-3)
Supplement: Supplementary file 6 — Percentages of sites with CB values less and greater than 1 for different types of R-M systems and phage genomes. (PDF 306 kb) [file 12864_2018_5324_MOESM6_ESM.pdf]

Table S1. Percentages of Type I sites with CB value less or greater than 1.

Genome type	Experimental dataset		Control dataset 1		Control dataset 2	
	CB < 1	CB > 1	CB < 1	CB > 1	CB < 1	CB > 1
dsDNA[1]	19454 (52.4%)	17523 (47.2%)	1123894 (51.2%)	1061083 (48.3%)	259161 (52.8%)	229040 (46.7%)
ssDNA[1,2]	880 (65.5%)	453 (33.7%)	28202 (58.9%)	19364 (40.5%)	109131 (62.0%)	65822 (37.4%)
ssRNA[2]	166 (65.1%)	89 (34.9%)	2595 (62.6%)	1531 (37.0%)	554576 (55.1%)	445131 (44.2%)
dsRNA	8 (47.1%)	9 (52.9%)	2283 (53.9%)	1934 (45.7%)	17811 (56.3%)	13598 (43.0%)

[1]The experimental set significantly differs from Control set 1 (*p*-value<0.01, Fisher's exact test)

[2]The experimental set significantly differs from Control set 2 (*p*-value<0.01, Fisher's exact test)

Table S2. Percentages of Type IIM sites with CB value less or greater than 1.

Genome type	Experimental dataset		Control dataset 1		Control dataset 2	
	CB < 1	CB > 1	CB < 1	CB > 1	CB < 1	CB > 1
dsDNA[2]	517 (53.6%)	447 (46.4%)	31033 (55.5%)	24826 (44.4%)	7490 (49.1%)	7716 (50.6%)
ssDNA[2]	74 (70.5%)	31 (29.5%)	1485 (57.7%)	1083 (42.1%)	6833 (46.4%)	7840 (53.2%)
ssRNA	11 (50.0%)	10 (45.5%)	123 (49.8%)	123 (49.8%)	19128 (51.8%)	17674 (47.9%)
dsRNA	1 (100.0%)	0 (0.0%)	50 (38.5%)	80 (61.5%)	851 (51.0%)	812 (48.6%)

Table S3. Percentages of Type IIG sites with CB value less or greater than 1.

Genome type	Experimental dataset		Control dataset 1		Control dataset 2	
	CB < 1	CB > 1	CB < 1	CB > 1	CB < 1	CB > 1
dsDNA	2218 (50.0%)	2210 (49.8%)	532837 (50.3%)	522623 (49.4%)	135717 (50.8%)	130518 (48.8%)
ssDNA	210 (54.3%)	174 (45.0%)	20970 (53.6%)	17920 (45.8%)	94165 (55.8%)	73623 (43.6%)
ssRNA	33 (49.3%)	33 (49.3%)	2021 (54.0%)	1701 (45.5%)	328254 (51.9%)	300991 (47.6%)
dsRNA	0 (0.0%)	0 (0.0%)	1267 (51.3%)	1195 (48.4%)	12320 (53.3%)	10670 (46.1%)

Table S4. Percentages of Type III sites with CB value less or greater than 1.

Genome type	Experimental dataset		Control dataset 1		Control dataset 2	
	CB < 1	CB > 1	CB < 1	CB > 1	CB < 1	CB > 1
dsDNA[1]	3256 (51.7%)	3039 (48.2%)	240882 (49.4%)	246265 (50.5%)	66544 (50.0%)	66147 (49.7%)
ssDNA[1,2]	457 (63.0%)	265 (36.6%)	11120 (51.9%)	10232 (47.7%)	60383 (53.4%)	52143 (46.1%)
ssRNA	69 (51.5%)	65 (48.5%)	1103 (52.0%)	1009 (47.6%)	162116 (49.7%)	163084 (50.0%)
dsRNA	4 (66.7%)	2 (33.3%)	599 (52.7%)	535 (47.1%)	6769 (51.2%)	6404 (48.4%)

Table S5. Percentages of Type IV sites with CB value less or greater than 1.

Genome type	Experimental dataset		Control dataset 1		Control dataset 2	
	CB < 1	CB > 1	CB < 1	CB > 1	CB < 1	CB > 1
dsDNA[1,2]	477 (91.4%)	45 (8.6%)	4316 (70.9%)	1768 (29.1%)	855 (50.4%)	839 (49.5%)
ssDNA[2]	43 (84.3%)	8 (15.7%)	216 (74.0%)	76 (26.0%)	765 (44.0%)	970 (55.7%)
ssRNA	5 (50.0%)	5 (50.0%)	17 (60.7%)	11 (39.3%)	2290 (54.4%)	1911 (45.4%)
dsRNA	0 (0.0%)	0 (0.0%)	3 (21.4%)	11 (78.6%)	102 (52.6%)	92 (47.4%)

Table S6. Percentages of asymmetric Type II sites with CB value less or greater than 1.

Genome type	Experimental dataset		Control dataset 1		Control dataset 2	
	CB < 1	CB > 1	CB < 1	CB > 1	CB < 1	CB > 1
dsDNA[1,2]	3871 (57.5%)	2860 (42.4%)	224639 (51.6%)	209729 (48.2%)	58067 (50.5%)	56533 (49.2%)
ssDNA[1]	288 (60.4%)	185 (38.8%)	9490 (54.5%)	7841 (45.0%)	48212 (55.4%)	38396 (44.1%)
ssRNA	46 (50.5%)	43 (47.3%)	908 (53.2%)	789 (46.2%)	145262 (52.6%)	129583 (46.9%)
dsRNA	1 (50.0%)	1 (50.0%)	509 (50.7%)	491 (48.9%)	5616 (52.8%)	4979 (46.8%)
